# Isolated Cecal Pole Ischaemia: A Rare and Underrecognised Variant of Acute Mesenteric Ischaemia Mimicking Acute Appendicitis

**DOI:** 10.7759/cureus.93052

**Published:** 2025-09-23

**Authors:** Nay C Lin, Maria Iqbal, Ali A Warsi

**Affiliations:** 1 General Surgery, Furness General Hospital-University Hospitals of Morecambe Bay NHS Foundation Trust (UHMBT), Barrow-in-Furness, GBR

**Keywords:** appendicitis mimic, caecal ischaemia, ischemic colitis, right iliac fossa (rif) pain, vascular risk factors

## Abstract

Isolated caecal pole ischaemia is a rare and often underrecognised variant of acute mesenteric ischaemia (AMI) that can closely mimic the clinical presentation of acute appendicitis. This case report describes a 78-year-old man who presented with right iliac fossa pain and vomiting. Initial investigations suggested acute appendicitis; however, contrast-enhanced computed tomography (CT) revealed an ischaemic caecum and an occluded superior mesenteric artery (SMA). Despite conservative management, the patient’s condition deteriorated, and exploratory laparotomy revealed a gangrenous cecal pole, prompting ileocecal resection. Histopathology confirmed ischaemic necrosis, and the patient recovered well postoperatively. This case highlights the diagnostic challenges associated with isolated caecal pole ischaemia and underscores the importance of early imaging and surgical intervention, especially in patients with significant vascular risk factors.

## Introduction

Acute mesenteric ischaemia (AMI) is a critical condition characterised by inadequate blood flow to the intestines, leading to tissue injury ranging from mucosal ulceration to transmural infarction and bowel perforation [1].

Although AMI most commonly affects the small bowel and proximal colon, isolated involvement of the caecum, particularly the caecal pole, is a much rarer phenomenon [2]. Isolated caecal pole ischaemia presents a significant diagnostic challenge due to its nonspecific symptoms, which frequently resemble more common intra-abdominal conditions such as acute appendicitis [2].

Due to anatomical variation in its blood supply, the caecum may be particularly susceptible to ischaemic injury in the setting of systemic hypotension or localised mesenteric vascular compromise [3].

The lack of clear diagnostic criteria and underreporting of this condition contribute to frequent delays in diagnosis and management [4].

## Case presentation

A 78-year-old male presented to the Emergency Department with a 12-hour history of sharp, non-radiating lower abdominal pain. The pain was constant and worsened with movement. He had experienced multiple episodes of non-bilious vomiting but denied diarrhoea, rectal bleeding, or urinary symptoms. His medical history included type 2 diabetes mellitus and hypertension, for which he was taking amlodipine, lisinopril, metformin, and omeprazole. He was a long-term smoker, reporting a 50-pack-year history.

On examination, the patient was alert and hemodynamically stable. Abdominal palpation revealed marked tenderness and rebound in the right iliac fossa, consistent with localized peritoneal irritation. The remainder of the abdomen was soft, non-distended, and without signs of generalized peritonitis. Bowel sounds were present. Digital rectal examination was unremarkable.

Laboratory investigations (Table 1) revealed a markedly raised white blood cell count of 19.3 × 10⁹/L with neutrophils at 16.7 × 10⁹/L. C-reactive protein was also elevated at 57.1 mg/L, while procalcitonin was raised at 0.51 ng/mL (reference <0.5). Serum lactate was slightly increased at 1.5 mmol/L. Renal function, liver enzymes, and electrolytes were within normal limits. An electrocardiogram (ECG) performed on admission demonstrated sinus rhythm, with no evidence of atrial fibrillation.

**Table 1:** Laboratory investigations on admission to the surgical department

Test Items	Value	Units	Reference Range
Red blood cell (RBC) count	4.41	×10¹²/L	4.5 – 5.9
Haematocrit level	37.4	%	40.0 – 50.0
Haemoglobin level	135	g/L	130 – 170
MCH level	31.5	pg	27.0 – 32.0
Mean cell haemoglobin (MCH)	30.5	pg	27.0 – 32.0
Mean cell volume (MCV)	84.9	fL	77.0 – 100.0
Red blood cell distribution width	13.4	%	11.6 – 14.0
White Blood Cell count	19.3	×10⁹/L	4.0 – 10.0
Neutrophil count	16.7	×10⁹/L	2.0 – 7.5
Monocyte count	0.8	×10⁹/L	0.2 – 1.0
Lymphocyte count	1.5	×10⁹/L	1.0 – 3.0
Basophil count	0.10	×10⁹/L	<0.2
Eosinophil count	0.20	×10⁹/L	0.02 – 0.50
Platelet count	300	×10⁹/L	150 – 400
Sodium level	131	mmol/L	133 – 146
Chloride level	96	mmol/L	95 – 108
Potassium level	4.4	mmol/L	3.5 – 5.3
Urea level	3.8	mmol/L	2.5 – 7.8
Creatinine level	65	µmol/L	<120
Alanine aminotransferase (ALT)	20	Int Unit/L	0 – 50
Alkaline Phosphatase (ALP)	118	Int Unit/L	<120
Total protein level	71	g/L	60 – 80
Albumin level	42	g/L	35 – 50
Bilirubin level	12	µmol/L	<21
γ-glutamyl transpeptidase (GGT)	27	Int Unit/L	<50
Globulin level	27	g/L	22 – 40
Procalcitonin level	0.51	ng/mL	<0.5
C-reactive protein (CRP) level	57.1	mg/L	<10
APTT ratio	1.06	—	0.80 – 1.20
Derived fibrinogen level	4.4	g/L	1.5 – 4.5
INR	0.9	—	0.8 – 1.2
Serum lactate	1.5	mmol/L	0.5 – 1.6

A working diagnosis of acute appendicitis was made based on the clinical presentation. However, in view of the patient’s age and risk factors, a contrast-enhanced CT scan of the abdomen and pelvis was performed.

**Figure 1:**
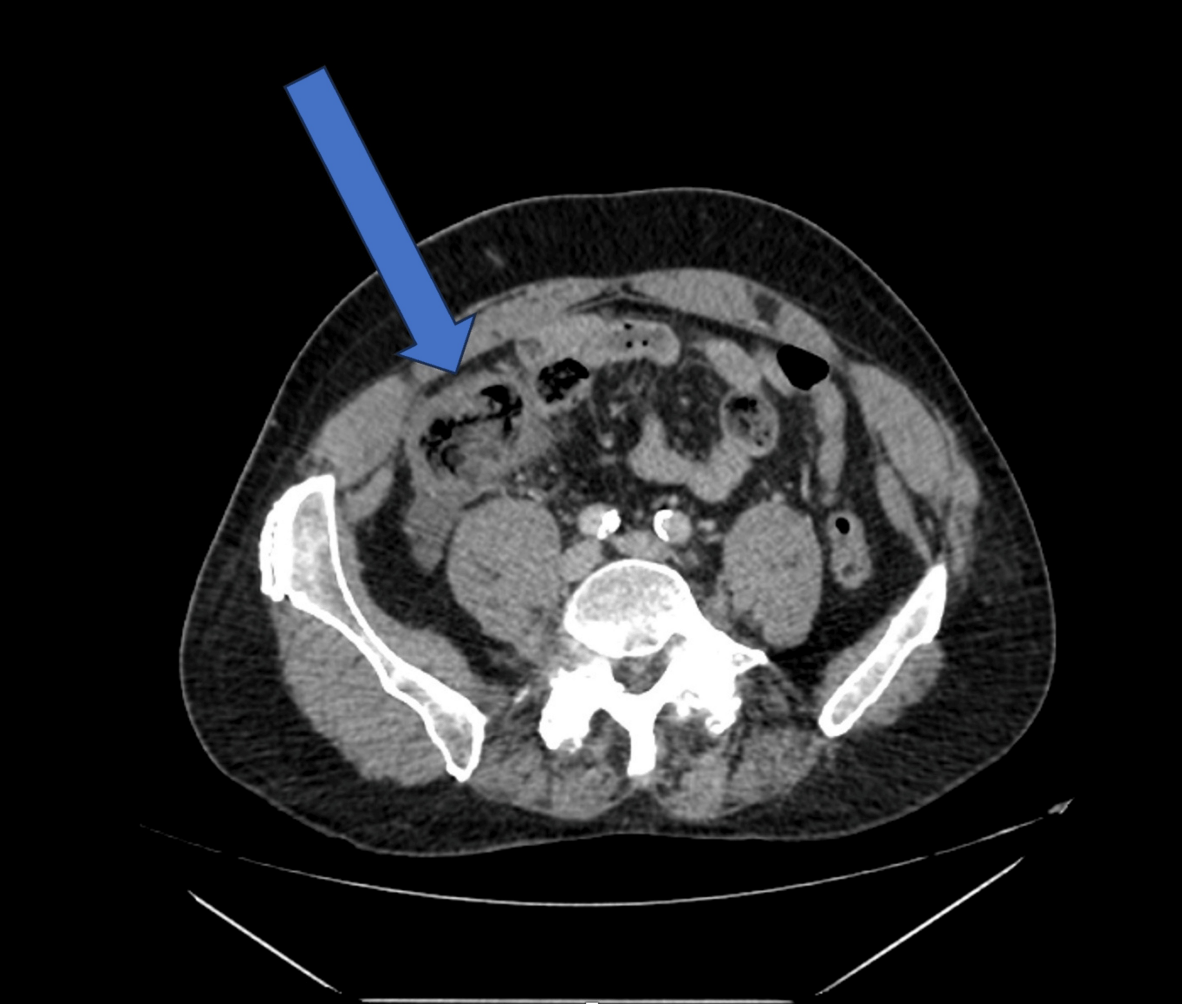
CECT abdomen and pelvis. The arrow highlights the cecal wall, which appears thickened and hypoenhancing with surrounding fat stranding, consistent with ischaemia CECT: Contrast Enhanced Computed Tomography

**Figure 2:**
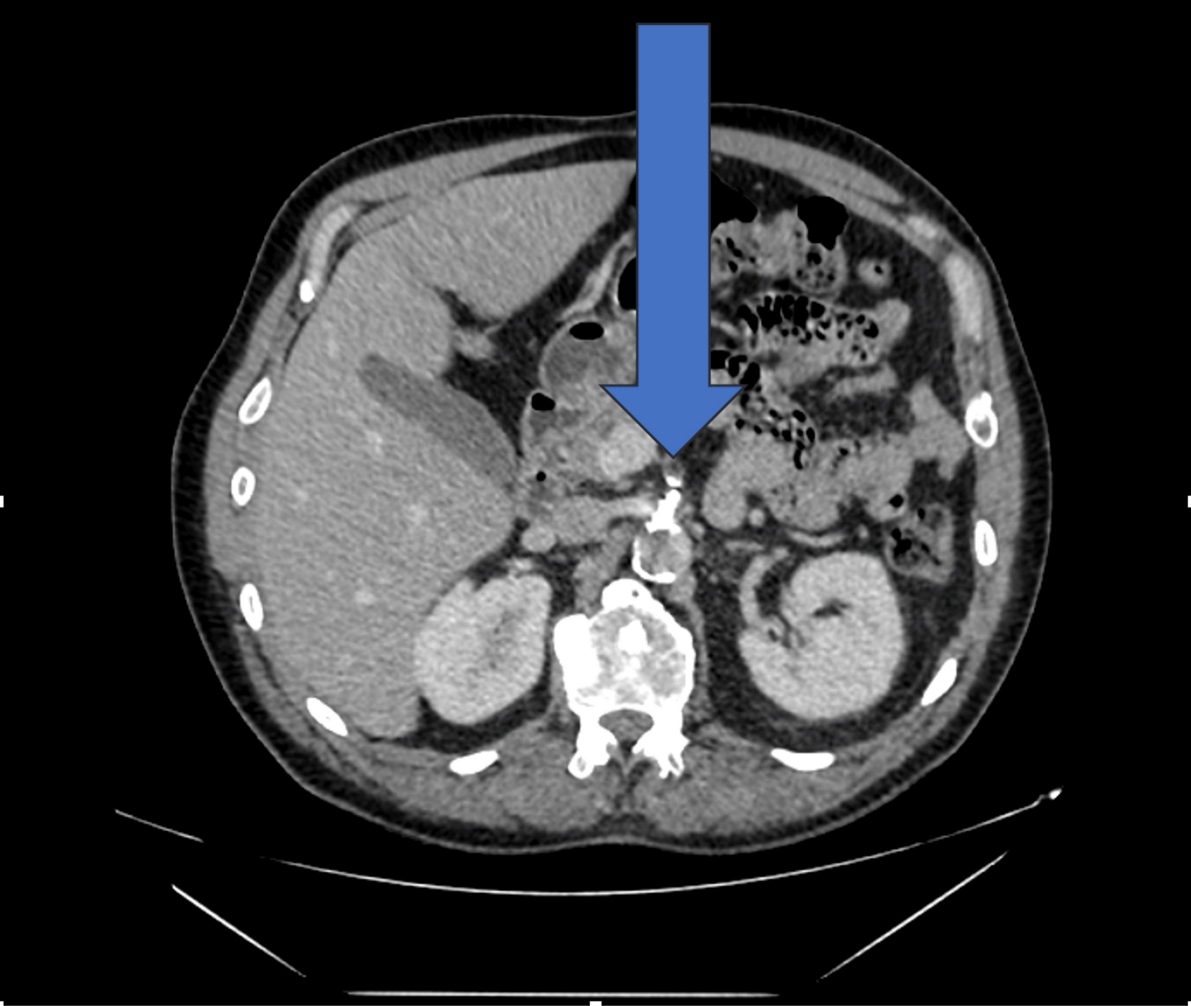
CECT abdomen and pelvis. The arrow indicates the proximal superior mesenteric artery, showing near-complete occlusion with a calcified, narrowed lumen CECT: Contrast Enhanced Computed Tomography

The contrast-enhanced CT scan of the abdomen and pelvis (Figure 1) revealed a markedly abnormal cecum with a thickened, shaggy wall and reduced contrast enhancement, consistent with ischemia. Surrounding inflammatory fat stranding and pericolic fluid were also noted. The appendix appeared normal. Importantly, the scan demonstrated significant calcific atherosclerosis and near-complete occlusion of the proximal superior mesenteric artery (Figure 2). The inferior mesenteric artery and celiac axis remained patent. There was no evidence of pneumatosis intestinalis, portal venous gas, or free intraperitoneal air.

The patient was admitted for conservative management and kept nil by mouth. He was commenced on intravenous fluids, broad-spectrum antibiotics, and, following vascular surgery input, therapeutic intravenous heparin. Despite this, over the subsequent 48 hours, the patient developed a temperature of 38°C and appeared increasingly unwell, although he remained hemodynamically stable. His abdominal tenderness worsened, and his inflammatory markers remained elevated.

At this stage, CECT abdomen and pelvis had already demonstrated caecal ischaemia with near-complete SMA occlusion, and the patient was clinically deteriorating. In view of the evolving peritonitis and the need for definitive management, an exploratory laparotomy was undertaken as the most appropriate intervention.

Intraoperatively, the caecal pole and lateral wall of the caecum were found to be gangrenous. The anteromedial caecum was relatively spared, and no evidence of bowel perforation was observed. There was turbid fluid in the right iliac fossa, but the terminal ileum and the rest of the colon appeared healthy. An ileocecal resection was performed, and a stapled primary anastomosis was constructed.

The patient was monitored in the intensive care unit postoperatively. Histopathological examination of the resected specimen revealed dusky, haemorrhagic, and partially necrotic caecum. Microscopy showed mucosal denudation, vascular congestion, crypt loss, and areas of mucosal necrosis and ulceration. No features of malignancy or chronic inflammatory disease were noted. Following stabilisation, the vascular team recommended the commencement of clopidogrel as secondary vascular prophylaxis. The patient’s recovery was uneventful, and he was discharged nine days after surgery with outpatient follow-up arranged with vascular surgery.

## Discussion

Isolated caecal pole ischaemia is a rare entity, representing a localised form of ischaemic colitis that often goes unrecognised due to its overlap with more common presentations such as acute appendicitis [5].

Ischaemic colitis primarily affects the left colon; however, right-sided colon involvement, including the caecum, can occur in approximately 10-26% of cases. Isolated caecal pole ischaemia, however, remains uncommon [2].

The lack of specific diagnostic criteria and overlap with more common conditions like appendicitis contribute to diagnostic delays [4].

While AMI generally affects the small bowel, the caecum can become ischaemic in both occlusive and non-occlusive states [1]. In this patient, significant atherosclerosis with proximal superior mesenteric artery (SMA) occlusion likely compromised flow to the ileocolic artery and its cecal branches, leading to localised ischaemia.

Occlusive ischaemia typically results from thromboembolism or atherosclerotic plaque involving the mesenteric arteries, while non-occlusive ischaemia may arise in the setting of systemic hypoperfusion, such as in shock, sepsis, or heart failure [1].

Our patient had multiple risk factors for occlusive disease, including type 2 diabetes mellitus, hypertension, and a long-standing smoking history.

The caecum can be anatomically predisposed to ischaemia due to variability in its segmental blood supply and limited collateral circulation in some individuals. It is typically supplied by the anterior and posterior caecal arteries, branches of the ileocolic artery, which itself arises from the superior mesenteric artery (SMA). In the absence of a complete anastomotic arcade between these branches, collateral flow may be reduced, increasing ischaemic risk, particularly during systemic hypotension or mesenteric vascular compromise. These anatomical features have led some authors to describe the caecum as a potential “watershed” zone [5].

Clinically, isolated caecal pole ischaemia frequently presents with right lower quadrant abdominal pain, sometimes accompanied by fever, nausea, vomiting, and leukocytosis, features indistinguishable from appendicitis [2].

In this case, the correct diagnosis was suspected only after CT imaging demonstrated an abnormal-appearing caecum with a normal appendix, along with vascular calcification and SMA occlusion.

Contrast-enhanced CT remains the initial investigation of choice for suspected colon ischaemia when renal function permits, capable of demonstrating bowel wall thickening, hypoenhancement, pericolic fat stranding, pneumatosis coli, and features of vascular compromise, including poor vessel opacification or mesenteric vessel thrombosis [6].

However, findings may be subtle in early disease, underscoring the need for a high index of suspicion [7].

Management of isolated caecal pole ischaemia depends on the extent and severity of ischaemic injury [1]. In non-transmural cases, supportive measures such as bowel rest, intravenous fluids, antibiotics, and anticoagulation may suffice [1].

However, progression to transmural necrosis, as seen in this patient, mandates surgical intervention [1]. In this context, laparotomy was preferred over laparoscopy, as the priority was definitive management, rapid assessment of bowel viability, and limited resection of the gangrenous caecum. The most appropriate operation is determined intraoperatively based on the viability of adjacent bowel segments [1,7].

In this case, an ileocecal resection with primary anastomosis was successfully performed.

Histology confirmed ischaemic colitis with mucosal necrosis and ulceration, consistent with vascular aetiology [8]. The absence of malignancy or inflammatory bowel disease supported the diagnosis of isolated vascular ischaemia.

Early postoperative recovery was aided by intensive care monitoring, and the patient was discharged with appropriate secondary vascular prevention.

## Conclusions

This case underscores the importance of considering ischaemic colitis, particularly isolated caecal pole ischaemia, in the differential diagnosis of right iliac fossa pain, especially in elderly patients with cardiovascular risk factors. Although rare, isolated caecal pole ischaemia can mimic appendicitis and delay appropriate management if not recognised. A detailed clinical assessment, prompt imaging with contrast-enhanced computed tomography, and multidisciplinary involvement, including vascular surgery, are crucial in identifying and managing this condition. Early surgical intervention in cases of transmural ischaemia can significantly improve outcomes and reduce the risk of serious complications such as perforation and sepsis.
